# The Impact of a Clinical Decision Support System for Addressing Physical Activity and Healthy Eating During Smoking Cessation Treatment: Hybrid Type I Randomized Controlled Trial

**DOI:** 10.2196/37900

**Published:** 2022-09-30

**Authors:** Nadia Minian, Mathangee Lingam, Rahim Moineddin, Kevin E Thorpe, Scott Veldhuizen, Rosa Dragonetti, Laurie Zawertailo, Valerie H Taylor, Margaret Hahn, Wayne K deRuiter, Osnat C Melamed, Peter Selby

**Affiliations:** 1 Nicotine Dependence Service Centre for Addiction and Mental Health Toronto, ON Canada; 2 Department of Family and Community Medicine University of Toronto Toronto, ON Canada; 3 Campbell Family Mental Health Research Institute Centre for Addiction and Mental Health Toronto, ON Canada; 4 Institute of Medical Sciences University of Toronto Toronto, ON Canada; 5 Department of Pharmacology and Toxicology University of Toronto Toronto, ON Canada; 6 Dalla Lana School of Public Health University of Toronto Toronto, ON Canada; 7 Applied Health Research Centre Li Ka Shing Knowledge Institute St. Michael's Hospital Toronto, ON Canada; 8 Department of Psychiatry University of Calgary Calgary, ON Canada; 9 Department of Psychiatry University of Toronto Toronto, ON Canada; 10 Schizophrenia Division Centre for Addiction and Mental Health Toronto, ON Canada; 11 Banting and Best Diabetes Centre University of Toronto Toronto, ON Canada

**Keywords:** smoking cessation, physical activity, healthy eating, clinical decision support system, Canada, diet, intervention, smoking, primary care, program, treatment, clinical decision support, health behavior

## Abstract

**Background:**

People who smoke have other risk factors for chronic diseases, such as low levels of physical activity and poor diet. Clinical decision support systems (CDSSs) might help health care practitioners integrate interventions for diet and physical activity into their smoking cessation programming but could worsen quit rates.

**Objective:**

The aims of this study are to assess the effects of the addition of a CDSS for physical activity and diet on smoking cessation outcomes and to assess the implementation of the study.

**Methods:**

We conducted a pragmatic hybrid type I effectiveness-implementation trial with 232 team-based primary care practices in Ontario, Canada, from November 2019 to May 2021. We used a 2-arm randomized controlled trial comparing a CDSS addressing physical activity and diet to treatment as usual and used the Reach, Effectiveness, Adoption, Implementation, and Maintenance framework to measure implementation outcomes. The primary outcome was self-reported 7-day tobacco abstinence at 6 months.

**Results:**

We enrolled 5331 participants in the study. Of these, 2732 (51.2%) were randomized to the intervention group and 2599 (48.8%) to the control group. At the 6-month follow-up, 29.7% (634/2137) of respondents in the intervention arm and 27.3% (552/2020) in the control arm reported abstinence from tobacco. After multiple imputation, the absolute group difference was 2.1% (95% CI −0.5 to 4.6; *F*_1,1000.42_=2.43; *P*=.12). Mean exercise minutes changed from 32 (SD 44.7) to 110 (SD 196.1) in the intervention arm and from 32 (SD 45.1) to 113 (SD 195.1) in the control arm (group effect: B=−3.7 minutes; 95% CI −17.8 to 10.4; *P*=.61). Servings of fruit and vegetables changed from 2.64 servings to 2.42 servings in the intervention group and from 2.52 servings to 2.45 servings in the control group (incidence rate ratio for intervention group=0.98; 95% CI 0.93-1.02; *P*=.35).

**Conclusions:**

A CDSS for physical activity and diet may be added to a smoking cessation program without affecting the outcomes. Further research is needed to improve the impact of integrated health promotion interventions in primary care smoking cessation programs.

**Trial Registration:**

ClinicalTrials.gov NCT04223336 https://www.clinicaltrials.gov/ct2/show/NCT04223336

**International Registered Report Identifier (IRRID):**

RR2-10.2196/19157

## Introduction

### Background

Smoking, low levels of physical activity, and poor dietary habits are highly prevalent and the three leading behavioral causes of death worldwide [1-4]. The concurrence of these risk factors [5-8] compounds the risks of developing chronic diseases [5,9]. Behavioral interventions by health care practitioners addressing these risk factors are potentially cost-effective [10,11]. Furthermore, improvements in one behavior can positively impact other risky behaviors [5,12,13]. For example, increasing physical activity can help reduce acute cravings and withdrawal symptoms when quitting smoking [14-17]. While the link between improving dietary habits and smoking cessation is less clear, improving dietary habits may prevent some postcessation weight gain. This can be a barrier to quitting smoking and maintaining abstinence [18-20]. Given these relationships, it is important to adopt a holistic approach to addressing risk behaviors [21].

Clinical decision support systems (CDSSs) are a promising resource to effectively support health care practitioners with the delivery of integrated evidence-based interventions to their patients [22-24]. A CDSS is an electronic application that can synthesize complex patient-specific information and present tailored recommendations to health care practitioners in real time [22-24]. They are frequently used in health care settings to help improve adherence to clinical guidelines, reduce treatment errors, and improve preventive care [25-30]. Moreover, a CDSS collects relevant data from different sources and presents them to the user in a central and easily accessible format. This is associated with improved efficiency and alleviates time burden during treatment planning [31]. Since many primary care offices use electronic medical records [32], CDSSs are also well-suited for seamless integration into existing workflows and allows for rapid and widespread scalability. We demonstrated that the addition of a CDSS in a smoking cessation program increased the likelihood that patients with at-risk drinking accepted an educational resource to reduce or abstain from alcohol consumption [33].

### Objective

The Smoking Treatment for Ontario Patients (STOP) program is a province-wide initiative that works in partnership with primary care settings across Ontario to provide tobacco users with up to 26 weeks of behavioral counseling and no-cost nicotine replacement therapy for smoking cessation. Health care practitioners at these organizations use the STOP portal, a web-based data collection and treatment management tool, to enroll their patients into the STOP program. However, an analysis of former STOP participants showed that 62% and 96% of STOP participants reported being below the Canadian guidelines for physical activity [34] and fruit and vegetable consumption [35], respectively. The STOP portal currently has a built-in CDSS to guide health care practitioners with addressing depressive symptoms and at-risk alcohol use as part of smoking cessation treatment. Scaling up the STOP portal to incorporate an additional CDSS that encourages practitioners to address patients’ physical activity and diet as part of the overall smoking cessation treatment could provide an opportunity to improve smoking cessation rates. However, there are several potential risks and limitations with implementing CDSS, including alert fatigue and disruptions to the current workflow of health care practitioners [24]. While the CDSS is designed to streamline processes and promote integration, it may still have unintended negative consequences. For example, the CDSS may require clinicians to engage in additional steps to input data, which can disrupt their clinical workflow and reduce clinician time to treat the patient’s presenting complaint. The CDSS may also not be relevant for all patient populations or clinical encounters, and this could inadvertently introduce bias in treatment [36]. Since tobacco use is correlated with the largest reductions in health-adjusted life expectancy [1], a key part of the implementation of the CDSS for other modifiable risk behaviors should be to ensure that it does not negatively affect the likelihood of patients quitting smoking.

The aims of this study were to (1) assess whether adding a CDSS for physical activity and diet to a smoking cessation program positively or negatively affects smoking cessation outcomes and (2) assess the implementation of the study using the Reach, Effectiveness, Adoption, Implementation, and Maintenance (RE-AIM) framework [37]. The detailed protocol is described elsewhere [38]. In this manuscript, we report the quantitative findings.

## Methods

### Study Design

We conducted a pragmatic, hybrid type I effectiveness and implementation trial [39], which allowed for the simultaneous testing of intervention effectiveness and implementation feasibility in real-world settings. Health care practitioners in primary care settings often report barriers to offering comprehensive preventive health services, including not having enough time, skills, knowledge, or resources [40]. As a result, it is important to examine the effectiveness of the CDSS on patients’ behavior outcomes, as well as how the CDSS impacts health care practitioners’ ability to provide treatment to their patients. Examining both effectiveness and implementation outcomes can help to provide valuable insights into the uptake of the CDSS and provide context for any observed results. We measured the effectiveness of the CDSS in a smoking cessation program using a 2-arm randomized controlled trial comparing a physical activity and diet CDSS directed at practitioners (intervention) with treatment as usual (control). The RE-AIM framework [37] was used to measure implementation outcomes in the intervention group.

### Setting and Location

The trial was operationalized in team-based primary care practices in Ontario, Canada (family health teams [n=153], community health centers [n=61], and nurse practitioner–led clinics [n=18]), implementing the STOP program as of November 29, 2019.

### Preimplementation Measures

To better equip health care practitioners to implement the intervention, we undertook several knowledge translation initiatives based on the principles of the Interactive Systems Framework (ISF) for dissemination and implementation [41]. The ISF comprises three interacting systems that facilitate the implementation of research in real-world practice: delivery systems, synthesis and translation systems, and support systems [41]. In this study, the health care practitioners at the primary clinics acted as the delivery system. The ISF’s synthesis and translation systems were addressed by engaging STOP program participants in the cocreation of health behavior change messages and a self-monitoring resource for tracking their health behaviors [42]. As part of the ISF support system, we provided health care practitioners with training (via an interactive webinar [43]) around evidence-based recommendations for addressing physical activity and fruit and vegetable consumption as part of a smoking cessation treatment program [44].

### Extenuating Circumstances

Most studies were conducted during the COVID-19 pandemic. The COVID-19 pandemic state of emergency (SOE) was announced in Ontario on March 17, 2020 [45], which was approximately 4 months into the study. During this period, many of the primary care settings that implemented the STOP program transitioned to offering phone- and video-based appointments (eg, via phone or video) to their patients [45]. Consequently, practitioners had to virtually communicate any recommendations generated by the CDSS for physical activity and diet to patients. Therefore, we also examined how the pandemic may have affected the delivery of the intervention.

### Participants

Eligible participants were treatment-seeking cigarette smokers who enrolled in the STOP program at one of the partnering primary care settings and reported baseline physical activity levels [34] and fruit and vegetable consumption levels [35] that were lower than the national guidelines. Low levels of physical activity were defined as engaging in less than 150 minutes of moderate to vigorous exercise per week (Canadian Physical Activity Guidelines) [34]. Low levels of fruit and vegetable consumption were defined as consuming less than 7 servings (female) or 8 servings (male) of fruit and vegetables daily (2007 Canada’s Food Guide) [35]. Participants were also required to be English-speaking and provide at least one piece of contact information (email address or phone number) so that the study team could conduct follow-up surveys at 6 months following enrollment into the STOP program. Participants were enrolled in the STOP program through self-referral or practitioner referral. Assessment of whether a participant met the eligibility criteria (as listed above) for the study was determined using the patient’s self-reported responses to the corresponding questions in the STOP program’s enrollment survey. The enrollment survey was completed using the STOP program’s web-based portal (STOP portal). The eligibility criteria remained the same throughout the study and were not affected by changes in response to the COVID-19 pandemic.

### Treatment Arms

#### Intervention Arm

The intervention was a CDSS that alerted health care practitioners if their patient reported low levels of physical activity and fruit and vegetable consumption and provided recommendations for behavior interventions. The recommendations in the CDSS were based on the literature on the most effective types of behavior change techniques [44]. The CDSS first prompted the practitioners to provide a brief risk communication intervention for physical activity and fruit and vegetable consumption. The risk communication involved sharing information about the risk behavior and discussing how it would affect both the patient’s health and smoking cessation treatment. The practitioners were then prompted to provide (print or via email) the patient with a self-monitoring resource for these risk behaviors. The self-monitoring resource was a 1-page paper-based weekly tracking sheet that patients could use to record their smoking, physical activity, and fruit and vegetable consumption.

#### Control Arm

In the control arm, the CDSS did not alert practitioners to whether their patients reported low levels of physical activity and fruit and vegetable consumption and did not provide practitioner recommendations to address these risk behaviors. Health care practitioners experienced the STOP portal as usual, which includes a CDSS for depressive symptoms and alcohol use. Although the CDSS was not available in the control group, practitioners were not prevented from addressing physical activity and diet with their patients if they deemed it clinically appropriate. We did not track whether the practitioner provided any counseling to the control group.

### Outcomes

We used the RE-AIM framework to structure and interpret the study outcomes. The components of the RE-AIM framework are reach, effectiveness, adoption, implementation, and maintenance.

#### Reach

The reach of intervention was assessed by examining the changes in the proportion of enrollments that were recorded as having been completed directly on the portal, before and after the CDSS for physical activity and fruit and vegetable consumption were introduced.

Health care practitioners can administer the STOP baseline questionnaire using the portal or on paper. Given that the CDSS is only available to practitioners when they conduct the questionnaire using the portal, any decrease in this proportion could be an indication that practitioners were avoiding the intervention by switching to a paper enrollment.

#### Effectiveness

The primary outcome of interest for this study was self-reported smoking cessation (7-day point prevalence abstinence) at the 6-month follow-up following enrollment. This outcome was measured by a response of “No” to the following question: “Have you smoked a cigarette, even a puff, in the last 7 days?” Research comparing self-reported smoking status with a biochemical assessment of smoking has found that they are highly correlated [46-49]. The secondary outcomes were self-reported changes in physical activity levels and fruit and vegetable consumption levels between baseline to 6-month follow-up. The Exercise Vital Signs Screener [50] was adapted to assess changes in physical activity. This screener has been validated [50] and consists of two questions: “On average, how many days per week do you engage in moderate-to-strenuous (vigorous) exercise (like a brisk walk)?” “On these days, for how many minutes do you typically exercise at this level?” The responses to these 2 questions were multiplied to produce the total minutes per week of moderate-to-vigorous exercise. Fruit and vegetable consumption was measured using a single question: “In a typical day, how many total servings of fruits and vegetables do you eat? (One serving is 1/2 cup of fresh, frozen, or canned fruits or vegetables, or 1/2 cup of 100% juice. Please DO NOT include potatoes).”

At baseline (time of enrollment), these self-report questions were administered by a STOP practitioner. At the 6-month follow-up, we collected responses to these questions via phone, email, or during a visit to a practitioner. The threshold for significance for the primary and secondary outcomes was *P*<.05. This is the established standard in empirical research for determining statistical significance despite its limitations [51].

#### Adoption

Given that the intervention has several components and was personalized to meet the needs of the participants, we assessed how often each component was adopted. Specifically, we examined the proportion of participants in the intervention group who were offered a self-monitoring resource for physical activity and fruit and vegetable consumption by their health care practitioner when appropriate.

#### Implementation

To acquire an understanding of the degree of fidelity at which the intervention was implemented, we examined how many participants accepted the self-monitoring resources. The intervention was classified as fully implemented when eligible participants received the corresponding self-monitoring resources. The patient only had the opportunity to accept the resource if the practitioner made the decision to offer it to the patient.

#### Maintenance

To determine whether efforts to provide the intervention changed over time, we calculated the proportion of eligible enrollees in each month who were offered the self-monitoring resources. To examine the sustainability of the smoking cessation intervention, we calculated the proportion of participants who had stopped smoking at both the 6-month and 12-month follow-ups.

In our protocol, we had only outlined the patient-level outcome for our maintenance outcome. Upon reflection, we also included the outcomes at the setting level.

### Sample Size

We determined the sample size required to detect clinically meaningful differences in our primary outcome was 3998 participants (1999 per group). For this sample size calculation, the effect size was an absolute difference in proportions of 0.04, the standard in smoking cessation for clinical significance [52]. On the basis of the past STOP program 6-month follow-up data, the proportion of individuals who will quit at 6 months was estimated to be 0.26. The power was set to 80%, and α was set to .05.

On the basis of the STOP program’s follow-up completion rate in the years before this trial, we anticipated a loss to follow-up of 25%. This increased the necessary total sample size to 5331 (2666 per group).

**Figure 1:**
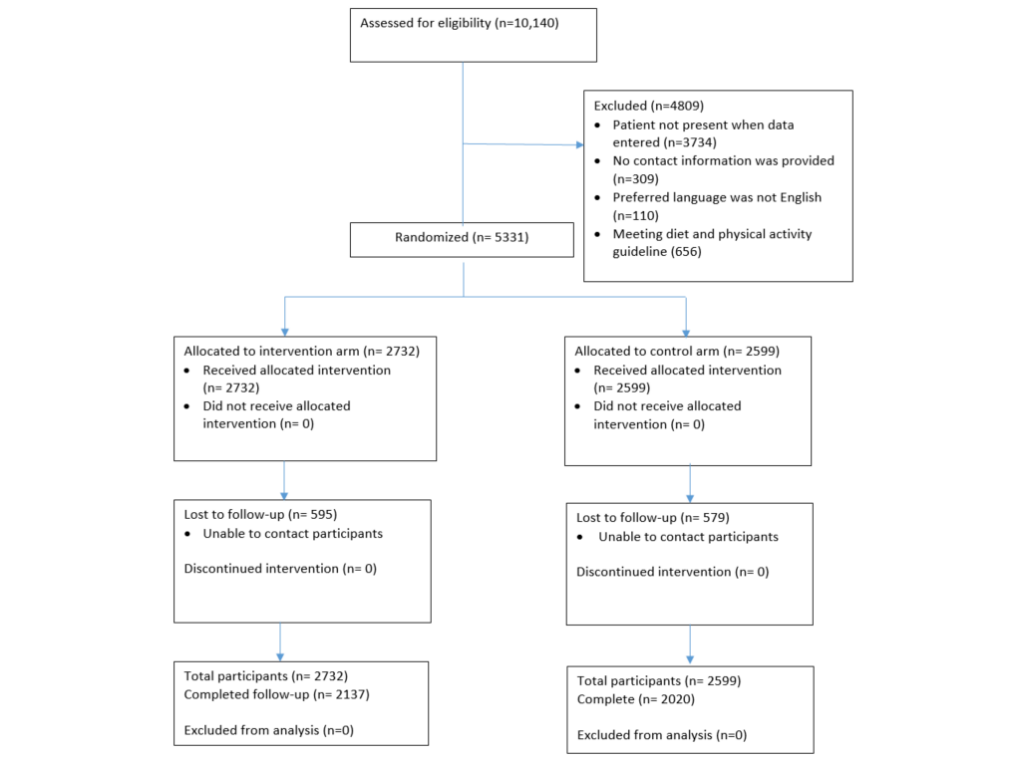
CONSORT (Consolidated Standards of Reporting Trials) flow diagram of the number participants allocated to intervention and control group and included in our primary and secondary data analyses.

As the randomization and intervention pathways were built into the STOP portal as part of the enrollment survey, we analyzed data when all participants in both arms had completed their 6-month follow-up. The analysis sample (Figure 1) included 4157 (control: n=2020, 48.6%; intervention: n=2137, 51.4%) participants who responded at the 6-month follow-up, as well as an additional 1174 participants who provided baseline and clinical data only (total sample, n=5331; control: n=2599, 48.8%; intervention: n=2732, 51.2%). Follow-up rates were 78% in both arms.

### Randomization

At the time of enrollment, we used a simple 1-to-1 randomization to assign eligible STOP participants to the intervention or control arm. The random allocation sequence was generated and implemented automatically by the STOP portal. The STOP portal performed the randomization using a built-in random number generator that determined, at the time of enrollment, which group the patient will be randomized to. The portal then automatically assigned the patient to the corresponding group. The practitioner was unable to override the allocation to the treatment arms. As health care practitioners treated both intervention and control patients, they were not blinded to the patient’s treatment allocation. However, patients were blinded to the study arm assignment.

### Statistical Analysis

Our analysis followed the principle of intention-to-treat, in which all participants were analyzed in the groups to which they were randomized. As specified by the study protocol, we tested differences in our primary outcome (the proportion of participants abstinent from cigarettes at the 6-month follow-up) using a chi-square test and differences in our secondary outcomes by regressing the follow-up measure on the baseline value and the study group. We used linear regression for the total weekly minutes of physical activity and negative binomial regression for the daily servings of fruits and vegetables. For these secondary outcomes, we only included participants who received each specific pathway (diet: control, n=2529 [47%]; intervention, n=2618 [49%]; exercise, control; n=1747 [33%]; intervention, n=1879 [35%]).

To minimize possible bias, we retained participants with missing baseline variables or follow-up data in our analysis. To address missing data, we used multivariate imputation by chained equations. In our multiple imputation models, we included variables capturing demographic and socioeconomic characteristics, heaviness of smoking, health conditions, self-rated importance of quitting and confidence in the ability to quit, the type of nicotine replacement therapy provided at the initial clinical contact, the number of clinical visits attended, and quit status at other time points (the most recent clinical visit and the 3-month and 12-month follow-ups). A total of 50 imputed data sets were generated. We used the mitools package in R 3.6 [53,54] to produce a chi-squared statistic with multiple imputation data sets. This produced an F-distributed D2 statistic.

#### Per-Protocol Analysis

The intervention comprised an automated alert to practitioners; not all patients in the intervention arm were offered the self-monitoring resources and not all patients who were offered the self-monitoring resources accepted them. To explore differences associated with the offering and acceptance of the resources, we conducted a secondary analysis in which we divided participants into four groups: the control arm, participant with whom practitioners conducted a risk communication discussion and offered the self-monitoring resources for these behaviors, participants receiving neither a risk communication discussion nor an offer of resources, and participants receiving either a discussion or an offer but not both. These analyses were similar to those described earlier but with 4 groups. If the participants were targeted with both interventions, we used the higher intensity level to determine their group.

#### Analysis of Reach Outcome

The CDSS operated only when practitioners reported that they were completing the baseline survey in the presence of the patient. To test whether the implementation of CDSS changed this reporting (as might be expected if clinicians were avoiding the pathway), we fit a piecewise logistic regression model of change over time, with a random intercept for the clinic. The COVID-19 pandemic also began during data collection; as a result, we fit a 3-part spline with two indicator variables, one for the beginning of the study and one for the beginning of the pandemic.

#### Sensitivity Analyses

To assess the effects of our treatment of missing data, we conducted a complete-case sensitivity analysis for our primary outcome by computing a chi-square statistic for the association between group and smoking cessation. To test for an effect of the COVID-19 pandemic on the effects of the study intervention, we fit an additional logistic regression model such as an interaction between the study arm and an indicator variable that was 1 if the enrollment took place after the SOE declaration and 0 otherwise.

### Ethics Approval

This study was approved by the Research Ethics Board of the Centre for Addiction and Mental Health (REB#:119-2018) and is registered at ClinicalTrials.gov (NCT04223336).

## Results

### Overview

Between November 2019 and October 2020, we enrolled 5331 patients with low levels of fruit and vegetable consumption or physical activity. Figure 1 provides a breakdown of the number of participants allocated to the intervention and control groups and included in our primary and secondary data analyses. We randomized 2599 (48.8%) participants to the control group and 2732 (51.2%) to the intervention group.

There were no major differences in the baseline characteristics of participants in the intervention and control groups (Table 1). Nearly all participants were daily smokers and over half have attempted to quit at least once in the year before their STOP enrollment. A little over a third of the participants reported being currently employed, and a quarter of the participants reported a household income below CAD $40,000 (USD $30,379.80).

In the intervention group, 853 (31%) participants reported low levels of fruit and vegetable consumption only, 114 (4%) reported low physical activity levels only, and 1765 (65%) reported both low levels of fruit and vegetable consumption and low physical activity levels. In the control group, 852 (33%) participants reported low fruit or vegetable consumption only, 70 (3%) reported low physical activity levels only, and 1677 (65%) reported both.

**Table 1:** Baseline characteristics of participants in study sample (n=5331).

Baseline characteristics				Intervention (n=2732)		Control (n=2599)		Total missing, n (%)		*P* value	
Age (years), mean (SD)				52.9 (13.9)		53.2 (13.7)		1 (<1)		.34	
Sex (female), n (%)				1464 (54)		1321 (51)		1 (<1)		.09	
First Nations, Metis, Inuit, n (%)				174 (7)		170 (7)		154 (3)		.78	
High school diploma, n (%)				1925 (75)		1800 (74)		321 (6)		.33	
Employed in past week, n (%)				982 (36)		882 (35)		82 (2)		.34	
Household income CAD ≥$40,000 (US $30,379.80), n (%)				377 (25)		371 (25)		2357 (44)		.78	
**Smoking**											
	Daily smoker, n (%)			2576 (94)		2430 (94)		3 (<1)		.24	
	Willing to set quit date, n (%)			1905 (70)		1808 (70)		0 (<1)		.92	
	Importance of quitting, mean (SD)			9.2 (1.2)		9.2 (1.2)		22 (<1)		.46	
	Confidence in ability to quit, mean (SD)			7.5 (2.0)		7.4 (2.0)		40 (<1)		.26	
**Past-year quit attempts**											.77
	None, n (%)			1273 (47)		1235 (48)		N/A^a^			
	≥1, n (%)			1441 (53)		1346 (52)		N/A			
	Missing			N/A		N/A		36 (1)			
**BMI (kg/m^2^)**											.79
	**Underweight (<18.5), n (%)**			77 (3)		70 (3)		N/A			
		Normal (≥18.5 and <25), n (%)	804 (32)		745 (31)		N/A				
		Overweight (≥25 and <30), n (%)	825 (33)		785 (33)		N/A				
		Obese (≥30), n (%)	786 (32)		780 (33)		N/A				
		Missing	N/A		N/A		459 (9)				
	At-risk drinking (AUDIT-C^b^), n (%)			967 (36)		899 (35)		82 (2)		.50	
	Low fruit or vegetable consumption levels, n (%)			2618 (98)		2529 (99)		106 (2)		.01	
	Low physical activity levels, n (%)			1879 (70)		1747 (69)		109 (2)		.34	
	At risk of depressive symptoms (PHQ-2^c^ score ≥3), n (%)			396 (15)		365 (15)		184 (3)		.62	
**Lifetime history of physical comorbid conditions**											
	Hypertension, n (%)			924 (34)		922 (36)		68 (1)		.18	
	High cholesterol, n (%)			889 (33)		875 (35)		125 (2)		.34	
	Heart disease, n (%)			393 (15)		376 (15)		88 (2)		.90	
	Stroke, n (%)			149 (6)		140 (5)		61 (1)		.94	
	Diabetes, n (%)			437 (16)		435 (17)		73 (1)		.47	
	COPD^d^, n (%)			808 (30)		734 (29)		152 (3)		.32	
	Rheumatoid arthritis, n (%)			199 (7)		200 (8)		140 (3)		.56	
	Chronic pain, n (%)			1050 (39)		1002 (39)		66 (1)		.91	
	Cancer, n (%)			264 (10)		245 (10)		73 (1)		.76	
**Lifetime history of psychiatric comorbid conditions**											
	Depression, n (%)			1149 (43)		1056 (41)		78 (1)		.28	
	Anxiety, n (%)			1191 (44)		1096 (43)		82 (2)		.31	
	Schizophrenia, n (%)			78 (3)		86 (3)		164 (3)		.34	
	Bipolar disorder, n (%)			184 (7)		166 (7)		94 (2)		.67	
**Lifetime history of substance use disorder**											
	Drug use disorder, n (%)			252 (9)		214 (8)		79 (1)		.21	
	Alcohol use disorder, n (%)			284 (11)		253 (10)		74 (1)		.39	
**Organization type**											.05
	Family health team, n (%)			2026 (74)		1856 (71)		N/A			
	Community health center, n (%)			622 (23)		666 (26)		N/A			
	Nurse practitioner–led clinic, n (%)			84 (3)		77 (3)		N/A			

^a^N/A: not applicable.

^b^AUDIT-C: Alcohol Use Disorders Identification Test.

^c^PHQ-2: Patient Health Questionnaire.

^d^COPD: chronic obstructive pulmonary disease.

### Reach

Our model for health care practitioner engagement with CDSS, which is a proxy for whether health care practitioners were avoiding the intervention by enrolling patients on paper instead of directly on the internet, showed that there was no change in the probability of reporting that a patient was present during enrollment following the beginning of the study (odds ratio [OR] 1.11, 95% CI 0.90-1.35; *P*=.33). However, this model also showed that this probability decreased sharply following the onset of the pandemic (OR 0.46, 95% CI 0.35-0.60; *P*<.001) but then increased (OR per month 1.46, 95% CI 1.38-1.54; *P*<.001). From May 2020 onward, the proportion of enrollments that were conducted directly on the internet was 68% (2518/3701), compared with an average of 58% (46,263/79,505) before the pandemic.

### Effectiveness

Of the participants responding at the 6-month follow-up, 27.3% (552/2020) in the control arm and 29.7% (634/2137) in the intervention arm were abstinent from tobacco at the follow-up. Our pooled estimates of proportions after multiple imputations were 25.9% (95% CI 24.2%-27.6%) and 28.0% (95% CI 26.1%-29.8%), respectively, corresponding to an absolute group difference of 2.1% (95% CI −0.5% to 4.6%). This difference did not meet our threshold for significance (*F*_1,1000.42_=2.43; *P*=.12). From baseline to the 6-month follow-up, the mean exercise minutes changed from 32 to 113 in the control arm and from 32 to 110 in the intervention arm (group effect: coef=−3.7 minutes, 95% CI −17.8 to 10.4; *P*=.61). The large apparent overall increase in exercise minutes is likely due to regression toward the mean resulting from the use of a cutoff point at baseline. For servings of fruit and vegetables, group means changed from 2.52 at baseline to 2.45 at 6 months in the control group and from 2.64 to 2.42 in the intervention group (incidence rate ratio for intervention group 0.98, 95% CI 0.93-1.02; *P*=.35).

### Adoption

In the intervention group, 1765 participants reported both low levels of fruit and vegetable consumption and low physical activity levels. Of these participants, 1083 (61%) were offered both physical activity and self-monitoring resources for fruit and vegetable consumption. Of the 853 participants who reported low levels of fruit and vegetable consumption (but met the physical activity guidelines) 526 (62%) were offered the self-monitoring resource for diet. Of the 114 participants who reported low physical activity levels (but met the nutrition guidelines) 66 (58%) were offered the self-monitoring resource for physical activity.

### Implementation

Of the 1765 intervention group participants who were offered the appropriate self-monitoring resource for physical activity or fruit and vegetable consumption, 624 (37%) accepted at least one self-monitoring resource.

### Maintenance

The proportion of participants in the intervention group who received an offer of one or both interventions was 67% (932/1402) during the pre–COVID-19 pandemic period; this declined to 60% (791/1329) after the pandemic began in Ontario in March 2020 (difference between these 2 periods: *χ*^2^_1_=14.2, *P*<.001).

Monthly proportions during the pandemic period did not differ beyond chance variation (*χ*^2^_6_=3.0; *P*=.81), and we did not find evidence of a linear trend (point-biserial correlation=−0.02; *P*=.51). Of the 552 control participants who achieved smoking cessation at 6 months, 438 (79%) responded to a 12-month follow-up. Of these, 322 (74%) were abstinent from smoking at 12 months. In the intervention arm, 634 participants had quit at 6 months. Of these participants, 507 (80%) responded at 12 months and 372 (74%) were not smoking at 12 months.

### Per-Protocol Analysis

In the intervention group, 757 (27.7%) patients received neither a risk communication discussion nor an offer of self-monitoring resource, 810 (29.6%) received one or the other, and 1165 (42.6%) received both. The quit proportions were 27.5% (95% CI 24.0%-31.0%) for those receiving neither resource, 27.6% (95% CI 24.1%-31.0%) for those receiving 1 resource, and 28.5% (95% CI 25.8%-31.3%) for those receiving both resources. The variability in outcomes across the 4 per-protocol levels (including the control arm) was not significant (*F*_3,1289.78_=0.956; *P*=.41).

### Sensitivity Analyses

Reanalyzing data using only complete cases showed, similar to our main analysis, that the group difference in tobacco abstinence at the 6-month follow-up did not meet our criterion for significance (intervention group: 634/2137, 29.7%; control group: 552/2020, 27.3%; *χ*^2^_1_=2.8; *P*=.10). The intervention effect also did not differ for people enrolling after the beginning of the COVID-19 pandemic (test for interaction: *z*=0.58, *P*=.56).

## Discussion

### Principal Findings

The addition of a CDSS for physical activity and fruit and vegetable consumption to a smoking cessation program did not negatively affect 6-month smoking cessation outcomes and did not negatively impact the reach of the smoking cessation program. However, it did not have a significant impact on participants’ physical activity or fruit and vegetable consumption at 6 months. That said, we saw that health care practitioners adopted the intervention (offered a self-monitoring resource to eligible participants) with approximately 60% (1083/1675) of their participants. Among the participants who were offered a resource, 37% (624/1675) accepted it. Of the participants who had quit at 6 months and who answered the 12-month survey, 74% (372/507) remained smoke-free, regardless of the study arm.

Given that most of this study took place during the initial phases of COVID-19 pandemic, where many primary care sites partially closed for nonurgent matters at the start of the lockdown and then transitioned to offering virtual services [55], it was important to examine how the pandemic may have impacted the delivery of the intervention. Our analysis showed that the proportion of baseline assessments being completed using the portal decreased sharply at the beginning of the pandemic but increased a couple of months later, exceeding the prepandemic proportion. Our analysis also showed that, compared with before the pandemic, a smaller proportion of participants received a physical activity or diet intervention.

### Interpretation and Comparison With Prior Work

Taken together, these results indicate that we can modify a smoking cessation program to be more holistic without negatively impacting smoking cessation, the single most important behavior change for reducing chronic disease-related mortality [52]. While there is ample evidence that modifying multiple health behaviors improves population health and reduces health care expenditures [56,57], there is insufficient research on effective ways to implement these changes. Furthermore, when multiple health behavior changes are necessary, knowing the impact of changing one behavior on another is important for health care practitioners as well as for decision-makers. For example, some research findings show that when physical activity and diet interventions were added to smoking cessation programs, there is a reduction in smoking [58,59]. However, other studies have reported either an adverse effect or no effect when physical activity and diet was integrated into a smoking cessation programming [60].

The results also showed that health care practitioners adopted the intervention (offered a brief intervention or self-monitoring resource) with approximately 60% (1083/1765) of their patients who were eligible, and 37% (624/1765) of these patients accepted the resource. The adoption outcome for this intervention was considerably higher than what we have seen for alcohol use in the STOP program, 21% of patients who drank alcohol above guidelines were offered an educational resource [33], and elsewhere for other health behavior interventions [61]. The implementation outcome (acceptance of resource by patient) in this trial was also higher than the ones reported for delivering a mood management intervention [61,62].

### COVID-19 Pandemic

The results of this study were likely affected by the onset of the COVID-19 pandemic. As we mentioned earlier, most of this study took place during the COVID-19 pandemic, which affected the context in which this study occurred, including the outer setting, the inner setting, and the health care practitioners and the participants’ behavior. From previous studies, we know that there were significantly fewer new enrollments and subsequent visits to the STOP program during the initial phase of the pandemic and that there was an increased number of STOP participants who reported being unemployed, as well as having substance use, mental health, and physical health diagnoses [55]. We also know that in the STOP program, the likelihood of successful smoking cessation after treatment dropped during the pandemic [63]. All of these factors might have affected both the implementation and effectiveness outcomes. For example, practitioners might have been less likely to recommend eating more fruit and vegetables if a participant reported that they were recently unemployed but more likely to recommend physical activity, which can be done without incurring a financial cost. Practitioners were also impacted by greater time constraints related to COVID-19 pandemic (ie, redeployment) and may not have been able to spend as much time addressing physical activity and diet with their participants [45].

While there was a large drop in the use of the portal to complete STOP enrollments during the SOE, within a couple of months the proportion of enrollments using the portal exceeded the prepandemic proportions (68%, compared with an average of 58% before the pandemic). This may be indicative of the positive impact the virtual care can have on a patient’s treatment experience, including minimizing logistical burdens around appointment management [64]. Since the appointments can be conducted over the phone, practitioners may have found it convenient to do the enrollment directly on the portal, instead of having the patient complete it on paper.

### Strengths and Limitations

This study had several strengths: it tested a simple web-based CDSS to facilitate the delivery of an intervention to address physical activity and diet among people who smoke. The results are generalizable given that we tested it in a large geographical area, with sites offering different types of primary care with varying staffing models and patient populations (family health teams, community health centers, and nurse practitioner–led clinics). By conducting a hybrid effectiveness-implementation trial, we showed that the addition of the CDSS did not negatively affect the reach of the program or the effectiveness of smoking cessation and that it was adopted by health care practitioners. However, the results also show that there is room for improvement with respect to implementation increasing the likelihood of practitioners offering self-monitoring resources to their patients.

There are a few limitations that need to be acknowledged. Our primary and secondary outcomes use self-reported measures, which may not accurately represent behavior changes in patients. Historical self-reported outcomes rely on patients accurately remembering their current behavior; thus, there is an increased risk of error and bias. However, these self-reported measures have been validated, and there is no reason to believe that any self-report bias would differ between the intervention and control groups. This study excluded French-speaking participants. We might have reduced the representativeness of our sample by excluding French-speaking participants; however, French-speaking participants make up a very small proportion of the STOP program, and we do not believe that their responses to the intervention would have differed from the included patients.

The fruit and vegetable question includes 100% fruit juice as part of the servings, which is not in line with the latest Canada’s Food Guideline [65], which was published after this study was created. However, we were only looking for overall changes in fruit and vegetable consumption as an indicator that could be used in primary care and differences between the intervention and control groups. In terms of limitations at the practitioner level, we have no way of verifying whether the practitioners in the intervention group actually acted on the CDSS recommendations and guidance as intended. Furthermore, while patients were blinded to their treatment allocation, health care practitioners were not, as we used patient-level randomization. Health care practitioners treating the control group participants could still address physical activity and diet with these participants. This lack of health care practitioner blinding increases the risk of group contamination as the health care practitioner could take their learnings from the intervention group patients and apply it to the control group participants. However, the physical activity and fruit and vegetable consumption self-monitoring resources in the CDSS were only available to participants in the intervention group, which minimized the risk of any cross-contamination. Thus, future studies should consider including additional process measures to accurately track any potential cross-contamination in the control group. This can be as simple as routinely asking control group practitioners whether they provided their own intervention to the patient.

### Conclusions

The introduction of a CDSS that guides health care practitioners to address multiple health behaviors among their patients does not seem to affect smoking cessation success. Although additional research is needed, these findings demonstrate that the CDSS can be used to introduce holistic treatment approaches within a primary care smoking cessation program. Thus, the CDSS could be a potential solution to break with the siloed approach of behavior change, which has dominated many care treatment approaches.

## Appendix

Multimedia Appendix 1 CONSORT-EHEALTH checklist (V 1.6.1).

## Abbreviations

CDSS: clinical decision support system
ISF: Interactive Systems Framework
OR: odds ratio
RE-AIM: Reach, Effectiveness, Adoption, Implementation, and Maintenance
SOE: state of emergency
STOP: Smoking Treatment for Ontario Patients
